# Ultra-slim flexible bronchoscopy-guided topical hemostatic drugs administration for the management of life-threatening refractory pulmonary hemorrhage in a preterm infant: Case report

**DOI:** 10.3389/fped.2022.981006

**Published:** 2022-10-18

**Authors:** Yan Lin, Hong-fang Zhao, Meng-hua Xue, Bing-jie Xie, Ling-chao Zeng, Xun Jiang

**Affiliations:** ^1^ Department of Pediatrics, Tangdu Hospital, Air Force Medical University, Xi’an, China; ^2^ Department of Thoracic Surgery, Tangdu Hospital, Air Force Medical University, Xi’an, China

**Keywords:** pulmonary hemorrhage, preterm infant, refractory, HFOV, flexible bronchoscopy

## Abstract

Pulmonary hemorrhage (PH) is a rare acute catastrophic event with high mortality among neonates, especially preterm infants. Primary treatments included pulmonary surfactant, high-frequency oscillatory ventilation, epinephrine, coagulopathy management, and intermittent positive pressure ventilation. However, there are still challenges in diagnosing and treating refractory or focal pulmonary hemorrhages. Ultra-slim bronchoscopy has been widely used in the field of critically ill children and is increasingly being done in neonates with critical respiratory disease in recent years. In this study, we report a case with refractory pulmonary hemorrhage in premature infants, which was finally diagnosed as localized hemorrhage in the upper left lobe and cured by ultra-slim bronchoscopy-guided topical hemostatic drug administration. Bronchoscopy is an optional, safe, and practicable technique for early diagnosis and direct injection therapy of neonatal PH in managing life-threatening PH.

## Introduction

Pulmonary hemorrhage (PH) is a rare, life-threatening complication in premature infants with respiratory distress syndrome. The incidence of PH is 3 to 11 per 1,000 live births with PH occurring most commonly within the first few days of life (1–5). PH is typically seen in neonates weighing less than 1500 g, who often have a patent ductus arteriosus (PDA), infection complication, trauma, have been treated with surfactant, and are ventilated (4, 6, 7). Mortality rates of over 50% have been reported in premature infants (8), and as a result, PH does significantly increase the risk of later pulmonary or neurodevelopmental disabilities among those who survive (9, 10). There are several effective methods of managing PH in neonates, including surfactant, high-frequency oscillatory ventilation (HFOV), epinephrine, coagulopathy management, intermittent positive pressure ventilation, cocaine, and tolazoline (4). However, it remains challenging to diagnose and therapy precisely in refractory or focal PH cases. Here, we present a case of refractory PH in a preterm infant diagnosed by ultra-slim flexible bronchoscopy. Furthermore, video bronchoscopy provides accurate information about PH location and bronchoscopy-guided local hemostatic treatment. We aimed to provide a potentially effective treatment for refractory pulmonary hemorrhage in neonates.

## Case report

A 1-h-old male neonate was born to an Asian mother through cesarean delivery at 33^+3^ weeks of gestation due to severe preeclampsia, weighed 1,870 g, and had Apgar scores of 9, 10, and 10 at 1, 5, and 10 min, respectively. The infant was artificial insemination-assisted reproduction due to the mother's tubal obstruction.

**Figure 1:**
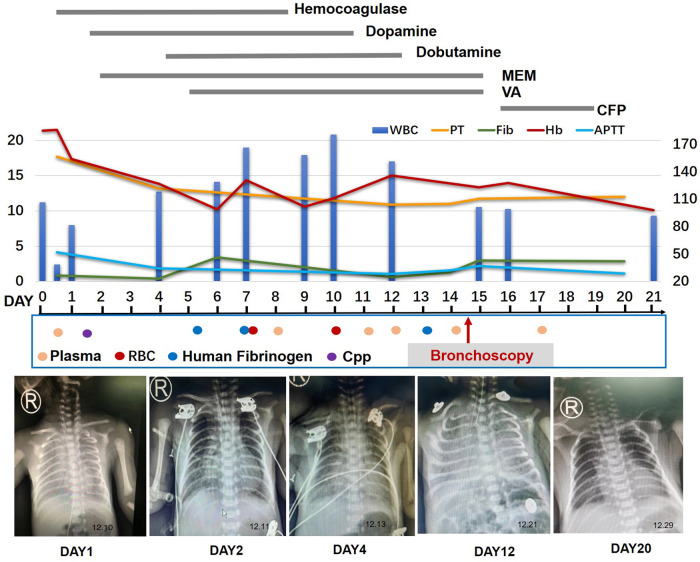
Timeline of the patient's clinical course and outcome. MEM, meropenem; VA, vancomycin; VOR, voriconazole; CFP, cefoperazone; RBC, red blood cells; Cpp, cryoprecipitate.

The infant presented with symptoms of respiratory distress, moaning, and a few pink foamy secretions from the mouth after birth. Physical exam revealed low breath sounds and pulmonary moist rales. The infant was diagnosed with neonatal respiratory distress syndrome (NRDS) and continuos positive airway pressure (CPAP)-assisted ventilation was administered immediately (FiO_2_ 25% PEEP 5 cmH_2_O), then the infant's condition gradually stabilized. However, 5 h later, plenty of blood gushed from the infant's mouth. Meanwhile, respiratory distress was notable for the respiratory rate of 60 per minute and SPO_2_ sat down to 82%. Therefore, we actively carried out endotracheal intubation and ventilator-assisted ventilation (synchronized intermittent mandatory ventilation (SIMV): FiO_2_ 40%, positive end expiratory pressure (PEE) 6.0 cmH_2_O, peak inspiratory pressure (PIP) 16 cmH_2_O, I: E 1:1.5). Simultaneously, epinephrine hydrochloride 1:10,000 and hemocoagulase were administered intratracheally. His complete blood count was 11.24 × 10^9^/L (with 80% neutrophils and 14.1% lymphocytes), Hb 185 g/L, and PLT 249 × 10^9^/L. Arterial blood gas analysis was PH 7.33, PaCO_2_ 40.1 mmHg, PaO_2_ 34 mmHg, HCO_3_ 19.6 mmol/L, and lactate 4.09 mmol/L, whereas his C-reactive protein levels were normal. FiO_2_ was increased from 40% to 60% after administration of vitamin K1, plasma, hemocoagulase, cefotaxime sodium, and penicillin. One hour later, the infant's breathing was gradually stabilized, and calf pulmonary surfactant was administered. However, 2 h later, large amounts of blood were again gushing out of the endotracheal tube. In addition to the above treatment, we switched to HFOV [Drager NV500: FiO_2_ 100%, mean airway pressure (MAP) 12 cmH_2_O, frequency 11 Hz, amplitude 20 cmH_2_O]. An ultrasonic cardiogram showed patent ductus arteriosus (3.3 mm) and patent foramen ovale (2.2 mm), and a chest x-ray showed exudative changes in both lungs. On day 2, the infant had dyspnea and respiratory distress under high-frequency ventilator-assisted, mottled skin, purpura, lethargy, abdominal distention, and capillary refill time (CRT) > 3 s. Simultaneously, the white blood cell count decreased to 2.4 × 10^9^/L (with 85% neutrophils and 13.3% lymphocytes), and a chest x-ray showed increased inflammation in the lower lobes of both lungs. In addition, arterial blood gas analysis was PH 7.32, PaCO_2_ 29.3 mmHg, PaO_2_ 54 mmHg, HCO_3_^−^14.5 mmol/L, and lactate 4.4 mmol/L, and his C-reactive protein levels increased to 42.47 mg/L. Therefore, meropenem was administered as further anti-infective treatment. On day 3, his white blood cell count increased to 12.74 × 10^9^/L (with 69.9% neutrophils and 16.2% lymphocytes), and Hb and PLT decreased to 127 g/L and 70 × 10^9^/L, respectively. Therefore, the antibiotic was gradually upgraded to vancomycin based on the infant's symptoms and the laboratory examination results. On day 5, the coagulation series of the infant seriously deteriorated, especially Fib, from 0.85 g/L (with prothrombin time (PT) 17.7 s, prothrombin activity (PTA) 42.9 s, activated partial thromboplastin time (APTT) 52 s, international normalized ratio (INR) 1.7, thrombin time (TT) 22.4 s, fibrinogen degradation products (FDP) 44.8 mg/L, and D-Dimer 13.43 mg/L) to 0.39 g/L (with PT 13.2 s, PTA 69.9 s, APTT 34.3 s, INR 1.2, TT 26.7 s, FDP 32.17 mg/L, and D-Dimer 3.78 mg/L). Therefore, human fibrinogen, cryoprecipitate (Cpp), plasma, and suspension red blood cells (RBCs) were administered. During the following 2 weeks, the infant continued to suffer from recurrent pulmonary bleeding to varying degrees with the assistance of a high-frequency ventilator (Figure 1).

**Figure 2:**
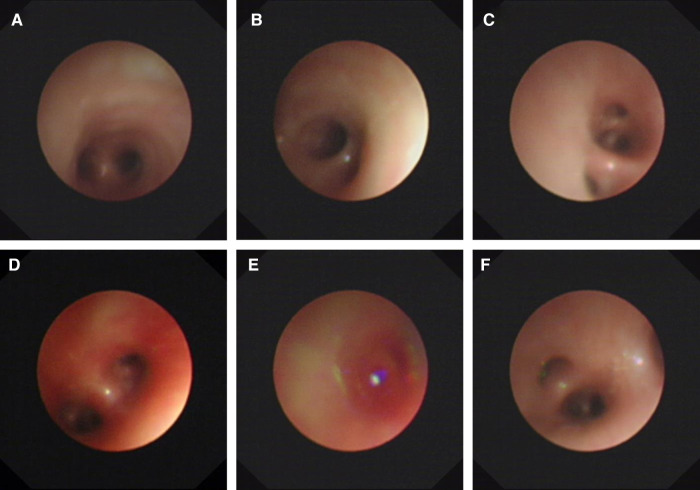
Ultra-slim flexible bronchoscopy showing pulmonary hemorrhage. (**A**) Carina. (**B**) Right main bronchus. (**C**) Right inferior lobar bronchus. (**D**) Left main bronchus. (**E**) Left superior lobar bronchus. (**F**) Left inferior lobar bronchus. Ultra-slim flexible video bronchoscopy showed a large outflow of fresh blood from the left upper lobe of the lung.

On day 14, the patient had recurrent symptoms of uncontrolled massive pulmonary hemorrhage, and vital signs were notable for the respiratory rate of 70 per minute and heart rate of 170 beats per minute. A physical exam revealed coarse breath sounds and pulmonary moist rales. The chest x-ray showed the range of high density of both lungs was larger than before, and the density increased. The dot and flake dense shadow, blurred boundary, and bronchial inflation sign were observed. With the informed consent of the parents, we performed an emergency ultra-slim flexible bronchoscopy (OLYMPUS BF-XP260F, outer diameter 2.8 mm, inner diameter 1.2 mm). Bronchoscopy entered the airway through the nose, pharynx, and larynx through the glottis. Furthermore, the trachea, right main bronchus, left main bronchus, right upper lobe, right middle lobe, right lower lobe, and left lower lobe were unobstructed with a small amount of blood (Figure 2). In addition, fresh blood was further observed in the upper lobe of the left lung. Therefore, hemostatic agents (adrenalin hydrochloride and hemagglutinin for injection) were injected after rapid suction and the removal of secretions from the upper lobe of the left lung. At the same time, a small number of deep secretions were collected under bronchoscopy and sent for etiological examination. The infant's respiration gradually stabilized after surgery, and we adjusted the ventilator mode to the non-invasive high-frequency ventilator (Drager NV500, FiO_2_ 50%, MAP 12 cmH_2_O, frequency 11 Hz, amplitude 23 cmH_2_O). On the second day, nucleic acid tests of 14 respiratory pathogens were negative, so the anti-infection treatment was changed to cefoperazone. Four days later, the infant was taken off the ventilator and started to stammer milk. *Informed consent was obtained from the infant's mother prior to the publication of this case report*.

## Discussion

The highest incidence of PH for preterm infants was 86.9 cases per 1,000 admissions at 24-week gestation, with a gradual decline at higher gestations. The incidence of PH for infants’ ≥32 weeks' gestation ranged from 0.6 to 1.9 cases per 1,000 admissions. Research has shown that the mortality is 40.6% within the first week after birth among infants <28 weeks' gestation (8), and the survivors have an increased risk of chronic lung disease and poor long-term outcomes (9, 10).

Risk factors for PH included severity of illness, intrauterine growth restriction, PDA, coagulopathy, the need for assisted ventilation, and a 10-min Apgar score (11). Accordingly, treatment of PH focuses on mechanical ventilation (especially HFOV), surfactant treatment, blood product (fresh frozen plasma and/or RBC), closure of PDA, nutritional support, analgesic, and sedative drugs (4, 12). Research has shown that HFOV is used to recruit compromised lungs, improve oxygenation through combined high MAPs and less tidal volumes, and eliminate carbon dioxide with few adverse cerebral side effects (13, 14). Therefore, HFOV was commonly used as the primary treatment method in PH. Ko et al. showed that 72% (13/18) newborn infants with PH responded to HFOV and survived (15), while 100% (6/6) survived without complications in the prospective observational study by Poddutoor et al. (16), although no cause for pulmonary hemorrhage was found in any of the infants (16). In this case, CPAP ventilation was administered after admission and adjusted to HFOV mode soon after an exacerbation. However, refractory pulmonary hemorrhage still occurred during HFOV application and when parameters were gradually decreased.

Pulmonary surfactant in the treatment of PH is controversial. On the one hand, PH appears to be a complication of surfactant therapy. In a meta-analysis, surfactant therapy was associated with an increased risk of PH (relative risk (RR) 1.47; 95% CI 1.05–2.07) indicating an increased risk for PH with surfactant therapy (17). In five multicenter, placebo-controlled trials of the synthetic surfactant in neonates, the incidence of clinical PH increased in those treated with the surfactant (18). These studies suggest that surfactant therapy may be a contributing factor, causing PH by inducing a rapid lowering of intrapulmonary pressure, which facilitates left-to-right shunting across a PDA and an increase in pulmonary blood flow (19). On the other hand, a prospective randomized controlled trial showed that natural surfactants improved oxygenation when administered for pulmonary hemorrhage in preterm infants (20). Amizuka et al. found that 21 of 26 neonates treated with single-dose surfactant 3.0 ± 1.3 h after the onset of hemorrhagic pulmonary edema exhibited a favorable response to exogenous surfactant, which was defined as a ventilatory index <0.047 at 1 h after surfactant administration (21). Similarly, a retrospective case series by Pandit et al. found that all 15 infants treated with surfactant had an improved ventilatory index and arterial/alveolar ratio (22). However, no recommendation for clinical practice based on randomized controlled trials can be presented. In our case, as a premature infant, the symptoms of respiratory distress appeared immediately after birth. Therefore, NRDS was diagnosed by combining relevant examinations, and CPAP-assisted ventilation was administered. However, 5 h after birth, the symptoms of respiratory distress worsened, and pulmonary hemorrhage occurred, so pulmonary surfactant (Ke-li-su, China) was administered according to the recommendation of the 2019 European NRDS Guidelines (23). Since the infant still had symptoms of respiratory distress and repeated pulmonary hemorrhage on the next day, porcine lung surfactant (Curosurf, Italy) was administrated 16 h after birth, considering its better solubility, while we found only about 3–4 h of symptom relief after every administration. Therefore, we believe that pulmonary surfactant has an adjunctive effect, while the efficacy for active bleeding is limited, and further research should be conducted.

Research has shown that PDA is a risk factor for the development of pulmonary hemorrhage in preterm infants (8, 24–26). Currently, PH occurs in 3%–5% of preterm ventilated infants with severe RDS who often suffer PDA and have received surfactant. The cause of PH is thought to be due to the rapid lowering of intrapulmonary pressure, which facilitates left-to-right shunting across a PDA and an increase in pulmonary blood flow (27). Kluckow et al. showed that infants with large PDA and early indomethacin treatment had significantly fewer early pH compared with infants who were not treated with indomethacin (28). These studies confirmed the importance of closure of the PDA in preterm infants with a high risk of PH. In this case, hemodynamically significant PDA (3.3 mm, left atrium/aortic root diameter = 1.5) was diagnosed by ultrasonic cardiogram. Symptomatic treatment, including fluid management (60–70 ml/kg), respiratory support, diuretics, and avoidance of hyperoxia, was administered first (29). One week later, the second echocardiography indicated that the size of the PDA was 2.2 mm. Considering the temporary absence of active hemorrhage, ibuprofen was administered as nasogastric tube therapy on the 8th day after birth. Although the PDA was closed 2 days later, repeated pulmonary hemorrhage still occurred.

Refractory pulmonary hemorrhage prompted further consideration of neonatal bronchoscopy to determine the etiology. Ultra-slim flexible video bronchoscopy is small and soft and enters below the bronchial segment and subsegment of the lung, which is beneficial to observe whether there are malformations or abnormalities of the tracheal mucosa and luminal, as well as whether there are excreta, foreign bodies, bleeding points, fistulas, and secretions, etc., and is widely used in pediatric clinical practice (30, 31). Due to the poor tolerance of small-weight airway stenosis to hypoxia and high technical requirements, there are few related studies in neonates. However, a recent European survey concerning practices in pediatric bronchoscopy showed the average number of bronchoscopies performed annually per center was 96, and 16% of those were performed in pediatric and neonatal ICUs. These results suggest that pediatric bronchoscopy has become more widely available and established. Atag et al. found that bronchoscopy assessments revealed at least one abnormality in 90.8% of patients whose median age was 5 months (range 0.3–205 months), and there were no major complications (32). Although bronchoscopy is increasingly used in the neonatal ICU, the diagnosis and treatment of pulmonary hemorrhage in a preterm infant have not been reported. In this case, HFOV, closed artery catheter, blood products, adrenaline, and antibiotics were administered systematically, and the infant still has repeatedly unexplained recurrent pulmonary hemorrhage. Therefore, with the family's informed consent, ultra-slim flexible bronchoscopy was performed, and local pressure administration of epinephrine and hemagglutinin was administered intraoperatively. Surprisingly, the symptoms of dyspnea were relieved quickly after the surgery.

In conclusion, PH is a life-threatening catastrophic event associated with a high mortality rate in neonates. Although traditional, conservative treatment is effective for the disease, some refractory PH is still difficult to treat clinically. Bronchoscopy is an essential tool for the diagnosis and management of respiratory disorders in NICUs and is a safe procedure in this vulnerable population. Endobronchial administration of hemostatics during bronchoscopy may be a safe and accurate option in the treatment of refractory PH. Further research is required for the follow-up of patients after bronchoscopy.

## Data Availability

The original contributions presented in the study are included in the article/Supplementary Material, further inquiries can be directed to the corresponding authors.
